# Disentangling the relative impacts of climate change and human activities on fluvial sediment supply to the coast by the world’s large rivers: Pearl River Basin, China

**DOI:** 10.1038/s41598-019-45442-2

**Published:** 2019-06-25

**Authors:** Roshanka Ranasinghe, Chuang Shou Wu, John Conallin, Trang Minh Duong, Edward Jamal Anthony

**Affiliations:** 1 Department of Water Science and Engineering, IHE Delft Institute for Water Education P.O. Box 3015 2610, DA Delft, The Netherlands; 2 Harbour, Coastal and Offshore Engineering, Deltares, PO Box 177, 2600 MH, Delft, The Netherlands; 3 Water Engineering and Management, Faculty of Engineering Technology, University of Twente, PO Box 217, 7500 AE, Enschede, The Netherlands; 4 Zhejiang Institute of Hydraulics & Estuary, Hangzhou, 310020 China; 5 Key Laboratory of Estuarine and Coastal Research in Zhejiang Province, Hangzhou, 030020 China; 6 Institute for Land Water and Society, Charles Sturt University, Albury - Wodonga, Elizabeth Mitchell Dr, Thurgoona, NSW 2640 Australia; 7 Aix Marseille Univ, CNRS, IRD, INRA, Coll France, CEREGE, Aix-en-Provence, France; 8 CNRS, UG, IFREMER, LEEISA USR 3456, Centre de recherche de Montabo, Cayenne, Guyane française France

**Keywords:** Civil engineering, Environmental impact

## Abstract

The world’s large rivers are under stress and experiencing unprecedented changes in hydrology, ecosystems, and fluvial sediment loads. Many of these rivers terminate at the great deltas of the world (home to 500 million people), which depend on fluvial sediments for their very existence. While fluvial sediment loads of large rivers have already been greatly modified by human activities, climate change is expected to further exacerbate the situation. But how does the effect of climate change on fluvial sediment loads compare with that of human impacts? Here, we address this question by combining historical observations and 21^st^ century projections for one of the world’s largest 25 rivers containing two mega dams; Pearl River, China. Our analysis shows that variations in fluvial sediment supply to the coast from the Pearl river over a ~150 year study period are dominated by human activities. Projected climate change driven 21^st^ century increases in riverflow will only compensate for about 1% of the human induced deficit in sediment load, leading to the coastal zone being starved of about 6000 Mt of sediment over the remainder of this century. A similar dominance of human impacts on fluvial sediment supply is likely at other heavily engineered rivers.

## Introduction

The flood plains and the deltaic coastal zones of the world’s large rivers, inhabited by some 2.7 billion people, have historically been the cornerstone of human civilization^1–6^. However, due to rapidly increasing demands for water, food, land and power, a number of human-induced stressors are resulting in large scale and potentially irrevocable changes in these natural systems^7–10^. One such change is the sustained erosion and ensuing land losses in the heavily populated deltas and adjacent coastlines (referred to hereon simply as *coastal zones* for convenience) due to the reduction of fluvial sediment supply to coast. More often than not, upstream dam constructions is the main driver of such reductions in fluvial sediment supply to the coast^11,12^. For example, the Three Gorges Dam in China has been retaining about 80% of sediment from upstream since it entered into operation in 2003, resulting in a massive reduction in fluvial sediment supply to the coast by the Yangtze river^13–15^. By 2005, more than 45,000 reservoirs over 15 meters high (i.e. mega dams) had been constructed across the world, which has trapped around 50% of the natural sediment load^16^ leading to dramatic decreases in fluvial sediment supply to the coast^11,12^.

Potential future land losses in coastal zones due to decreases in fluvial sediment supply is an increasing global concern due to the combination of (a) the significant increase in the construction of/plans for new mega dams over the last two decades^6,17–19^ (b) the continued growth of coastal communities (~10% of the global population)^20–25^, and (c) the eroding trend along at least 24% and up to 70% of the world’s sandy coastlines^26^. Moreover, although several studies have shown that fluvial sediment loads in large rivers have been more impacted by human activities than climate change over the 20^th^ century (and early 21^st^ century)^15,27–29^, projected accelerated climate change during the remainder of the 21^st^ century, and beyond^30^, is widely expected to affect riverflows and sediment loads in the world’s largest rivers in an unprecedented way^8,31–33^. Nevertheless, to date there is little quantitative understanding of how the relative contributions of climate change and human activities to fluvial sediment loads may evolve over the coming decades in many of the world’s important river systems. Such quantitative knowledge is however crucial to enable effective basin scale catchment and coastal zone management^29,34,35^. Here, we take a step towards addressing this knowledge gap by combining historical observations and 21^st^ century projections to quantify the relative contributions from both human impacts and climate change to the fluvial sediment supplied to the coast by the Pearl River, China over a ~150 year period (mid-20^th^ century - end of the 21^st^ century).

## Pearl river, china

**Figure 1:**
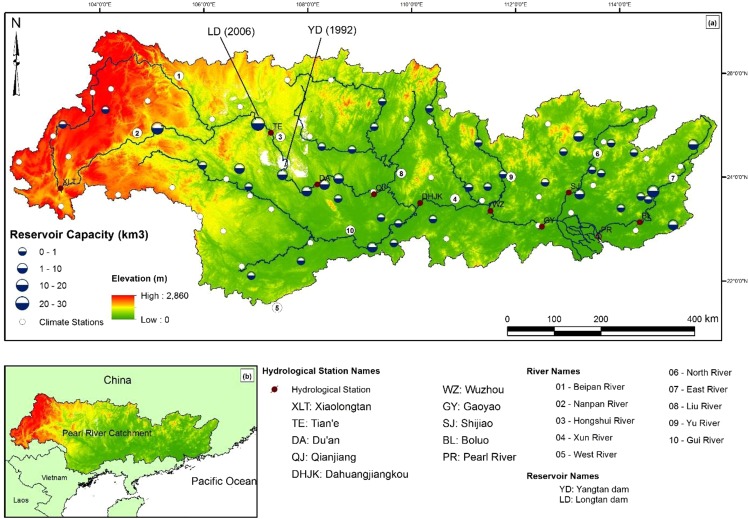
(**a**) Map of the Pearl River basin including elevation, tributaries and gauging stations; (**b**) Location of the Pearl River basin in China.

Pearl river, China, is the 2^nd^ largest river in China in terms of water discharge (after the Yangtze river), and being among the largest 25 rivers in the world, is a globally significant system^36–39^. The Pearl river basin (Fig. 1) is home to 230 million people^39^ and its catchment area spans 450,000 km^2^ with a catchment-wide average precipitation of 1470 mm/yr^38^. The average annual water discharge of the Pearl river is 280 million m^3^/yr and the sediment yield in the upper catchment is 71.5 Mt/yr^39^. The basin has been subjected to increased anthropogenic interventions since the 1980s due to the implementation of China’s open door policy and other reform policies such as the Great Western Development Plan^16^. Since the late 1980’s, the Pearl River delta has been subjected to massive land reclamations, leading to exponential urban and socio-economic growth^40^, resulting in a current population exceeding 50 million in the delta area alone^41^. The Pearl River contains two mega dams in its upper reaches: the Yangtan dam (110 m high with a maximum reservoir capacity of 3,432,000,000 m^3^; constructed 1985–1995) and the Longtan dam (216 m high with a maximum reservoir capacity of 27,270,000,000 m^3^; constructed 2001–2009), the latter being the world’s tallest gravity dam.

The Pearl River basin is a much studied system, with numerous previous studies focusing on diverse system characteristics such as the formation and evolution of the delta^42^, hydrological changes in response to human activities^43^, sediment trapping rates in dams^38,39^, delta shoreline changes^41,44–46^, estuarine engineering (e.g. sand excavation, reclamation, and dike building)^47^ and fluvial sediment loads^32,33,38,39,48^. However, all previous studies on the latter, which is the focus of this study, have concentrated on changes observed over the second half of the 20^th^ century and the first decade or so of the 21^st^ century. Here, for the first time, we investigate fluvial sediment loads in the Pearl River over the extended period 1954–2100; a period that not only encompasses marked historical changes caused by human activities, but also the future effects of projected accelerated climate change^30^. Specifically, here we analyse changes in fluvial sediment loads in the historical period 1954–2013, and derive future projections of fluvial sediment loads up to the end of the 21^st^ century under a worst case climate change scenario. The analysis of results provides quantitative insights on the relative contributions of climate change and human activities to variations in fluvial sediment supply to the coast over this ~150 year period spanning the past and the future.

### The past: 1954–2013

Monthly riverflow and sediment load data measured at 9 stations from 1954–2013 along the Pearl River (see methods and Fig. S1 in Supplementary Material) were used to gain insights on the spatio-temporal evolution of the fluvial sediment transport along the river. The measurement stations (XLT, TE, DA, QJ, DHJK, WZ, GY, SJ and BL) are shown in Fig. 1. To compute riverflow and sediment load at the Pearl river outlet (virtual station PR), the data at stations GY, SJ, and BL were added together.

Previous studies^33,38,39^ have indicated that, in terms of fluvial sediment loads in the Pearl river, the historical period from 1954 to 2013 can be divided into four discrete phases. It should be noted that while the exact timing of the four phases may be somewhat different among the West, North and East rivers of the Pearl river basin, Wu *et al*.^39^ have shown that the combined fluvial sediment load in the North and East rivers (SJ and BL) is about 10% of the total sediment supply to the coast (GY, SJ and BL combined). Therefore, as the focus of this study is the fluvial sediment supply to the coast from the Pearl river basin as a whole, here we have considered the same phase timings at all stations to enable the basin aggregated calculations that are necessary for this analysis.

**Figure 2:**
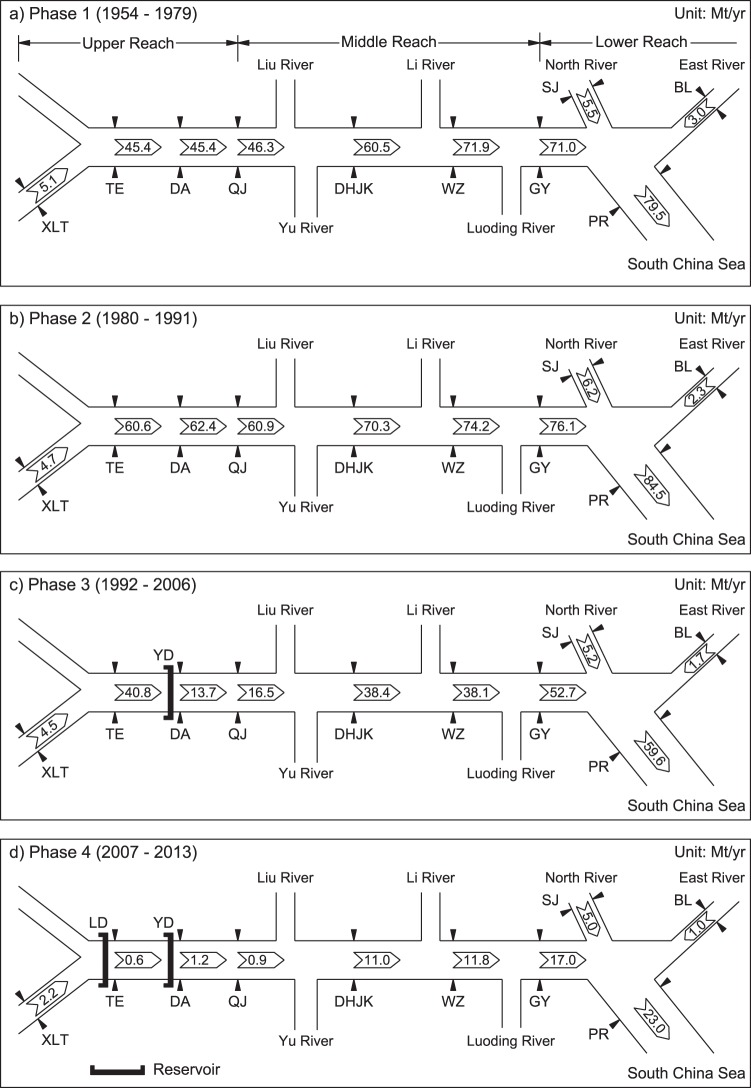
The spatio-temporal evolution of fluvial sediment loads along the Pearl river from 1954–2013. For station names, please see Fig. 1. YD and LD indicate Yangtan and Longtan dams respectively.

From the 1950s to the late 1970s, fluvial sediment loads in the Pearl river system have mainly been governed by river discharge, followed by an increased impact of deforestation till the end of the 1980s. From the early 1990s, dam construction has dominated the fluvial sediment loads in the Pearl River, with the period from the early 1990s to the mid-2000s being affected by the construction of the Yangtan dam, and the final period from the mid-2000s onward being affected by the construction of the Longtan dam. Following this philosophy, here we have divided the historical period 1954–2013 into the four phases shown in Fig. 2.

Analysis of the fluvial sediment loads along the main river reach (XLT to PR) during the four discrete phases shows distinct differences in the characteristics of sediment supply to the coast among the different phases. Over the period 1954–1979 (Phase 1), the sediment load increases along a downstream continuum with the upper reach (XLT to QJ) contributing over half of the total fluvial sediment supply to the coast, with further contributions from tributaries downstream. During the subsequent decade or so (1980–1991; Phase 2), fluvial sediment supply to the coast increases, mostly due to an increase in the contribution from the upper reach. From 1992–2006, the reverse behavior is observed where the fluvial sediment supply to the coast decreases below that during Phase 1. In the final period from 2007–2013 (Phase 4), fluvial sediment supply to the coast decreases even further to less than 30% that of Phase 1.

**Figure 3:**
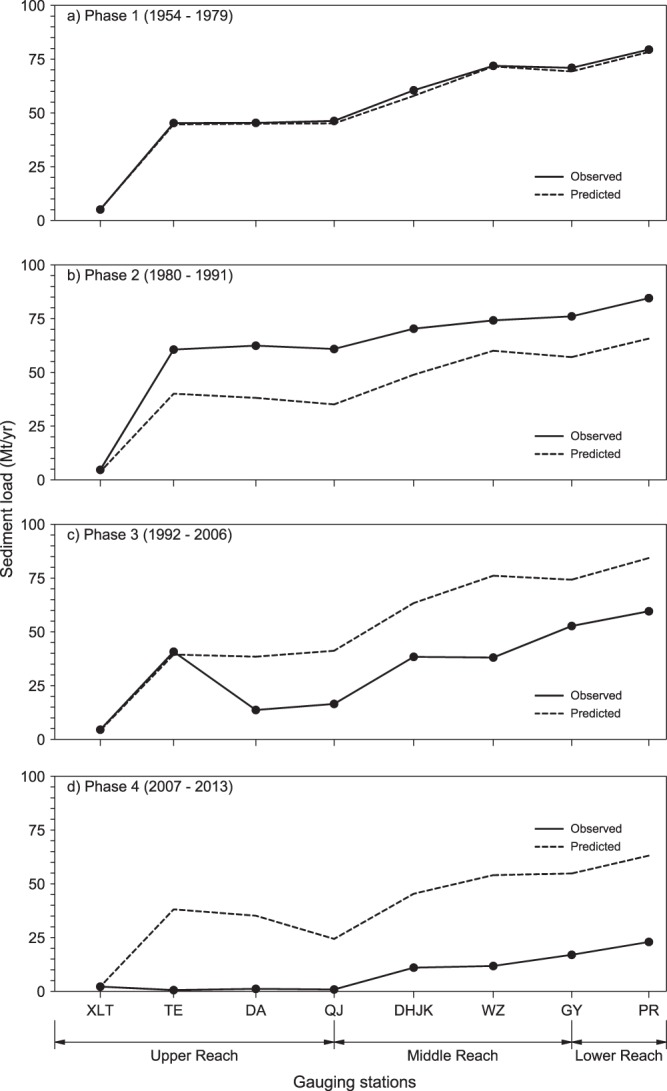
Comparison between sediment loads predicted using the Phase 1 rating curves (dashed line) and observed sediment loads along the Pearl River during the 4 different Phases.

To understand the physical drivers underlying the above described temporally varying fluvial sediment transport regime of the river, here we made strategic use of sediment rating curves (i.e. empirically determined relationships between sediment load and water discharge at measurement stations) developed for the 9 stations and the ocean outlet (PR) (see methods and Supplementary Material). During Phase 1 (1954–1979), the sediment load predicted by the rating curves (when used together with measured riverflows) follows the observed sediment load data at all 10 locations (Fig. 3a). Therefore, Phase 1 can be considered as a water discharge controlled phase (or a largely “natural” phase) devoid of significant human influences. Having established this “baseline”, it is then possible to use the Phase 1 rating curves to compute what the sediment loads “would have been” during the other 3 Phases in the absence of any significant human influences. It should however be noted that the baseline period, during which no significant human impacts are expected to have occurred, might vary among different reaches of the complex Pearl river system. However, along the main West river reach (XLT – PR), the sediment load is completely governed by river discharge over the 1954–1979 period (as shown by Fig. 3a), and hence this period is suitable as the overall baseline period for the purposes of this study.

Following the above hypothesis, the sediment loads predicted when using the Phase 1 rating curves together with measured monthly riverflows during Phases 2–4 were compared with the observed sediment loads during these Phases (Fig. 3b–d and Tables S1 and S2 in Supplementary material). Essentially, any departure of the computed sediment loads from that observed is indicative of non-natural behavior. Figure 3b shows that during Phase 2 (1980–1991), observed values at most stations exceeded the predicted sediment load along the main reach of the Pearl River basin, with an ultimate observed increase of 7% in fluvial sediment supply to the coast. This could be attributed to land use changes in the upper reaches due to Chinese economic reform policies which resulted in much of the area being cleared and utilized for agricultural production^40^. During Phase 3 (1992–2006), however, the observed sediment load sharply decreases compared to the predicted sediment load (Fig. 3c), with the fluvial sediment supply to the coast decreasing by about 25% compared to Phase 1. The main cause for this decrease in sediment load at the middle and lower reaches of the river is the construction of the Yangtan mega dam in the upper reach (indicated by YD in Fig. 2c). During Phase 4 (2007–2013) the sediment load decreases further compared to predicted sediment load (Fig. 3d), resulting in a massive 71% decrease in fluvial sediment supply to the coast compared to Phase 1. This is attributed to the placement of Longtan mega dam (indicated by LD in Fig. 2d) upstream of the Yangtan dam, trapping approximately 99% of the sediment input from the upper tributaries of the river^39^.

### Climate change versus Human impacts over 150 years (1954–2100)

For the purposes of this study, it is necessary to determine the relative contributions of climate change and human activities on fluvial sediment supply to the coast in a way that is consistent for both the past and the future (given that measured sediment loads are not available for the future). To that end, sediment loads predicted by the rating curves described above were here used together with reasonable assumptions. The computational procedure followed is illustrated in *Methods*.

**Figure 4:**
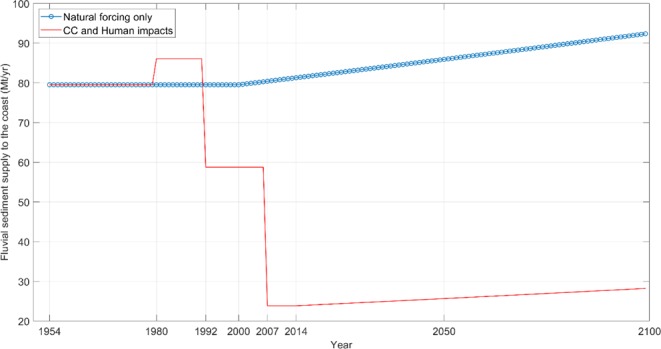
The fluvial sediment supply to the coast over the period 1954–2100 under the combined effect of climate change and human impacts (red line) and under natural forcing only, including climate change (blue circles).

First, the total fluvial sediment supply to the coast (i.e. at location PR) was computed under the combined effect of climate change and human activities. For Phases 1 to 4, the mean fluvial sediment supply at PR per Phase was computed using the monthly measured riverflow data and rating curves for PR (see Table S3 in Supplementary Material). Post-2014 riverflows were estimated using the 0–10% increase over the 21^st^ century projected for the Pearl River by Yan *et al*.^49^. Here, assuming a high end scenario, an increase of 10% in the runoff (by 2079–2099, relative to 1979–1999) was used in all calculations. The fluvial sediment supply for the period 2014–2099 was then calculated with the rating curve obtained for Phase 4 at location PR, with the assumption that no more significant human interventions will take place at this already heavily engineered system. The fluvial sediment supply to the coast under the combined effect of climate change and human impacts over the period 1954–2100 thus calculated is shown in Fig. 4 (red line).

Subsequently, the fluvial sediment supply the coast may have received due only to natural forcing (including climate change but excluding all human impacts) was computed. When all human impacts are neglected, ideally, the system can be expected to behave as it did during the “natural” Phase 1. Furthermore, several studies have indicated that climate change driven variations in runoff during the latter half of the 20^th^ century in the Pearl River basin are all but negligible^50^. Therefore, in this “natural forcing only” computation, the PR rating curve for Phase 1 was used together with the measured riverflows to compute the fluvial sediment supply to the coast for the period 1954–1999. For the entirety of the 21^st^ century, following the projections of Yan *et al*.^49^, an increase of 10% in the runoff (by 2079–2099, relative to 1979–1999) was adopted. In this 21^st^ century sediment load calculation too, the rating curve computed for the natural Phase (i.e. Phase 1) was used because all human impacts are being neglected in this calculation. The potential fluvial sediment supply to the coast under only natural forcing (including climate change) for the entire 1954–2100 period is shown in Fig. 4 (blue circles).

Figure 4 clearly shows the dramatic difference between what could have been (under natural forcing only) and the impact of human activities to date (assuming that no significant human interventions, positive or negative, will take place during the remainder of the 21^st^ century). The percentage reduction of fluvial sediment supply to the coast due to human impacts from 1954 to 2013 is 71%, as shown in Fig. 4. The projected CC driven increase in riverflow during the 21^st^ century will only compensate for 1% of this deficit by 2100.

Although a recent analysis of multi-decadal shoreline mobility in response to changes in fluvial sediment supply shows that the subaerial Pearl delta is still advancing seaward^51^ (largely due to coastal land reclamations^41^), other recent studies^52–54^ that have also examined the evolution of the sub-aqueous bathymetry of the Pearl delta have shown that parts of the delta are in fact presently eroding or accreting at a decreasing rate. Therefore, like several other large deltas in the world that have not yet been significantly or visibly impacted, the decrease in fluvial sediment supply to the coast identified here is likely to change the morphological evolution of the coastal zone adjacent to the Pearl River (inclusive of the Pearl delta) in the years ahead. For example, erosion of Yangtze delta is reported to have commenced when the fluvial sediment supply to the coast decreased by 50%^55^. Given that the computed decrease in fluvial sediment supply from 1954–2013 by the Pearl River is already 71%, it is therefore highly likely that the Pearl delta will also face a similar fate, sooner rather than later. Furthermore, sediment starvation by the river-adjacent coastline could lead to sustained coastline recession as has also been observed at the Yangze^55,56^, Ebro^57^ and Mekong rivers^26^. The projected decrease in fluvial sediment loads in the Pearl River could also have significant environmental implications, due to the associated reduction in the ability of the water column to self-decontaminate^38,48^. A potential positive impact of the projected decrease in fluvial sediment supply is increased nearshore zone primary productivity. Enhanced photosynthesis of planktons due to the potential increase in transparency of seawater associated with a reduction in suspended sediment concentration in the water column could result in such an increase in primary productivity in the nearshore zone^38,48^. This type of environmental impacts are not only relevant for the Pearl Delta, arguably one of the key economic regions in China, but also for nearby Macau and Hong Kong, and thus requires attention in future studies.

### Concluding remarks

The stability of river-adjacent coastal zones (inclusive of deltas) is strongly dependent on the continued supply of fluvial sediment to the coast. Any significant departure from the natural fluvial sediment supply is very likely to result in sustained erosion leading to large scale coastal zone land losses. With the strategic use of historical data and future projections, this study derived quantitative insights into the relative impacts of human activities and climate change on variations in fluvial sediment supply to the coast at the globally relevant Pearl River, China, over a ~150 year period (1954–2100).

This analysis showed that the fluvial sediment supply to the coast from the Pearl river has decreased (from its natural state) by 71% from 1954–2013 due to human activities, and that projected increases in riverflow due to climate change will compensate for just 1% of this deficit by the end of the 21^st^ century (if the impact of human activities is maintained at current levels). Based on these projections, the coastal zone of the Pearl River will be starved of approximately 6,000 Mt of fluvial sediment during the remainder of the 21^st^ century.

As in the case of the Pearl River, the dominance of human impacts over that of climate change on fluvial sediment supply to the coast is likely at other heavily engineered rivers around the world. If remedial measures are not implemented sooner rather than later, the result will be substantial sediment starvation along river-adjacent coastal zones leading to pervasive coastal erosion and ensuing land losses. This will not only threaten existing coastal zone communities and developments but will also compromise foreshadowed coastal zone developments and expansions.

Furthermore, the results of this study underline the importance of taking into account catchment effects, particularly those due to human activities, in coastal zone management and planning efforts which have historically looked at the coast mostly from the sea side (i.e. basing all decisions on characteristics of sediment transport driven by oceanic processes such as waves, tides, extreme sea levels, sea level rise etc.). The non-inclusion of a large human induced deficit in fluvial sediment supply to the coast, such as that projected here for the Pearl River, when considering the coastal zone sediment budget could lead to sub-optimal designs and decisions, which may, under some circumstances, place coastal settlements and communities at risk.

## Methods

### Data

Long-term water discharge and sediment load collected by the Pearl River Water Resource Commission, published data from the Bulletin of Chinese River Sediment (2003–2013)^58^ and the Bulletin of Guangxi Zhuang Autonomous Region River Sediment (2004, 2006, 2007)^59^ were used in this study. The annual averaged data are graphically shown in Figure S1 in Supplementary material.

### Development of sediment rating curves

Fitting a power function (Eq. 1) between fluvial sediment load (*Q*_*d*_) and water discharge (*Q*_*s*_) is a commonly used method to develop sediment rating curves for rivers^60–64^. From the empirical relationship between *Q*_*s*_ (km^3^ yr^−1^) and *Q*_*d*_ (Mt/yr) the following correlation can be established:

1 $${Q}_{d}=a.{{Q}_{s}}^{b}$$

where, *a* is the sediment rating coefficient and *b* is the rating exponent.

Extensive studies of this type of functions have found that the *a* coefficient is an index of erosion severity, or the source of sediment supply. A high *a* parameter is associated with intensely weathered material which can be easily eroded and transported^65^. The exponent *b* represents the erosive power of the river and the influence on the sediment supply from the entire catchment surface. The value of *b* can also be affected by the grain size distribution of the material available for transport in the river basin. Large values of *b* indicate an increase in sediment transport capacity of the river^62^. A detailed discussion on the spatio-temporal differences in the *a* and *b* coefficients as relevant to the Pearl river is provided by Zhang *et al*.^48^. The rating curve approach was exclusively used in this study to develop sediment rating curves for the 9 measurement stations and the virtual station at the ocean outlet of the river (PR). The *a* and *b* coefficient values thus obtained and the associated goodness of fit statistics are given in Table 1.

**Table 1:** Fitted rating curve parameters (a, b) and goodness of fit measures (r, p) at the 9 Pearl river measurement stations and the virtual station at the Pearl river inlet.

Station	Phase 1				Phase 2				Phase 3				Phase 4			
	*a*	*b*	*r*	*p*	*a*	*b*	*r*	*p*	*a*	*b*	*r*	*p*	*a*	*b*	*r*	*p*
XLT	0.63	1.43	0.95	<0.001	0.90	1.36	0.88	<0.001	0.84	1.24	0.89	=0.002	0.31	2.09	0.97	<0.001
TE	0.03	1.66	0.95	<0.001	0.12	1.58	0.68	<0.001	1.04	0.86	0.69	=0.05	6*10^−9^	1.56	0.98	>0.05
DA	0.03	1.78	0.94	<0.001	0.005	2.32	0.76	<0.001	1*10^−4^	2.02	0.70	=0.05	2*10^−8^	4.38	0.85	>0.05
QJ	0.02	1.88	0.82	<0.001	0.001	2.59	0.87	<0.001	8*10^−6^	3.41	0.79	<0.001	1*10^−8^	4.60	0.74	=0.001
DHJK	0.007	1.74	0.89	<0.001	0.0002	2.48	0.84	<0.001	4*10^−5^	2.64	0.80	<0.001	2*10^−5^	2.66	0.92	=0.005
WZ	0.005	1.78	0.91	<0.001	0.0006	2.24	0.81	=0.002	2*10^−4^	2.23	0.75	=0.002	2*10^−5^	2.58	0.75	=0.05
GY	0.008	1.68	0.92	<0.001	0.005	1.80	0.76	=0.005	4*10^−5^	2.60	0.89	<0.001	4*10^−4^	2.01	0.95	=0.001
SJ	0.01	1.61	0.95	<0.001	0.006	1.86	0.85	<0.001	0.0002	2.59	0.96	=0.001	0.001	2.27	0.88	<0.01
BL	0.05	1.33	0.98	<0.001	0.007	1.89	0.87	<0.001	0.01	1.57	0.91	<0.001	0.0002	2.69	0.99	<0.01
PR	0.009	1.59	0.86	<0.001	0.007	1.69	0.75	=0.005	2*10^−5^	2.58	0.91	<0.001	0.0009	1.84	0.96	<0.001

Phase 1: 1954-1979; Phase 2:1980–1991; Phase 3: 1992–2006; Phase 4: 2007–2013. Please see Fig. 1 for station locations.

### Computational procedure

**Figure 5:**
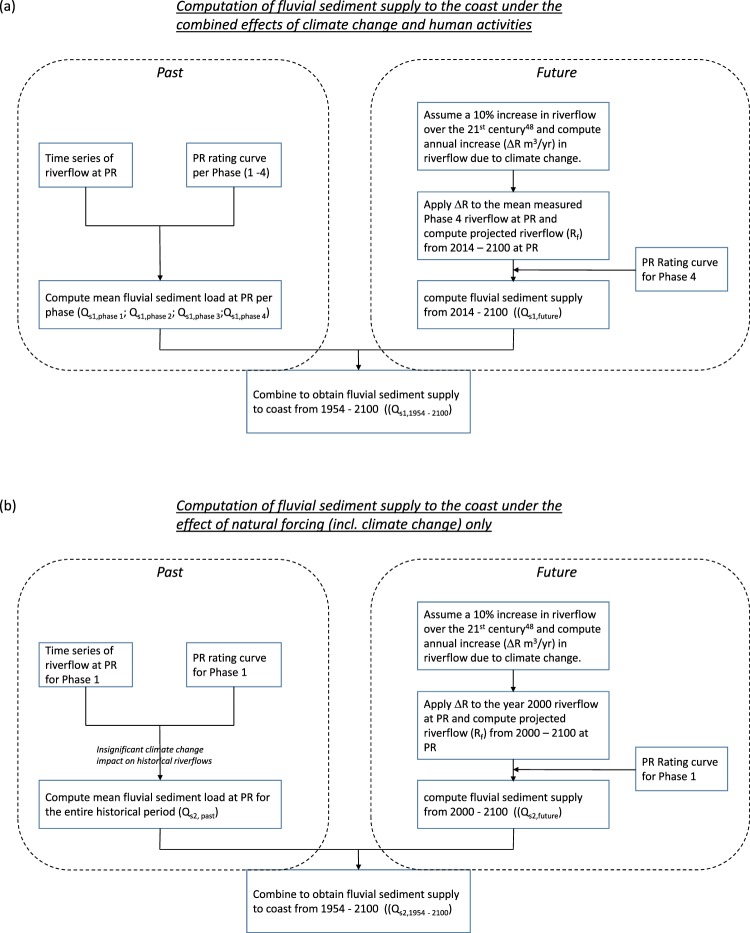
Computational procedure followed to calculate fluvial sediment supply to the coast over 1954–2100 under the effect of (**a**) both climate change and human activities, and (**b**) natural forcing (incl. climate change) only.

The procedures followed in this study to compute the fluvial sediment supply to the coast over the period 1954–2100 under the combined effect of climate change and human activities, and under natural forcing alone (including climate change effects) are shown in Fig. 5a,b respectively.

## Supplementary information

Supplementary information

## Data Availability

The datasets generated during and/or analysed during the current study are available from the corresponding author on reasonable request.
