# Supplementary material for: Disentangling the relative impacts of climate change and human activities on fluvial sediment supply to the coast by the world’s large rivers: Pearl River Basin, China
Source: Sci Rep. 2019 Jun 25;9:9236. doi: 10.1038/s41598-019-45442-2 (PMC6592904; doi:10.1038/s41598-019-45442-2)
Supplement: Supplementary file 1 — Supplementary information [file 41598_2019_45442_MOESM1_ESM.pdf]

SUPPLEMENTARY INFORMATION: Disentangling the relative impacts of climate change and human activities on fluvial sediment supply to the coast by the world's large rivers: Pearl River Basin, China.

Roshanka Ranasinghe[a, b, c], Chuang Shou Wu[d,e] , John Conallin[a, f], Trang Minh Duong[a,b], and Edward Jamal Anthony[g,h].

[a] *Department of Water Science and Engineering, IHE Delft Institute for Water Education, P.O. Box 3015 2610 DA Delft, The Netherlands*

[b] *Harbour Coastal and Offshore Engineering, Deltares, PO Box 177, 2600 MH Delft, The Netherlands*

[c]*Water Engineering and Management, Faculty of Engineering Technology, University of Twente, PO Box 217, 7500 AE Enschede, The Netherlands*

[d] *Zhejiang Institute of Hydraulics & Estuary, Hangzhou, 310020, China.*

[e] *Key Laboratory of Estuarine and Coastal Research in Zhejiang Province, Hangzhou 030020, China*

[f] *Institute for Land Water and Society, Charles Sturt University, Albury - Wodonga, Elizabeth Mitchell Dr, Thurgoona NSW 2640, Australia*

[g] *Aix Marseille Univ, CNRS, IRD, INRA, Coll France, CEREGE, Aix-en-Provence, France.*

[h] *CNRS, UG, IFREMER, LEEISA USR 3456, Centre de recherche de Montabo, Cayenne, Guyane française*

Corresponding author: Roshanka Ranasinghe: r.ranasinghe@un-ihe.org

SUPPLEMENTARY FIGURE (S1):

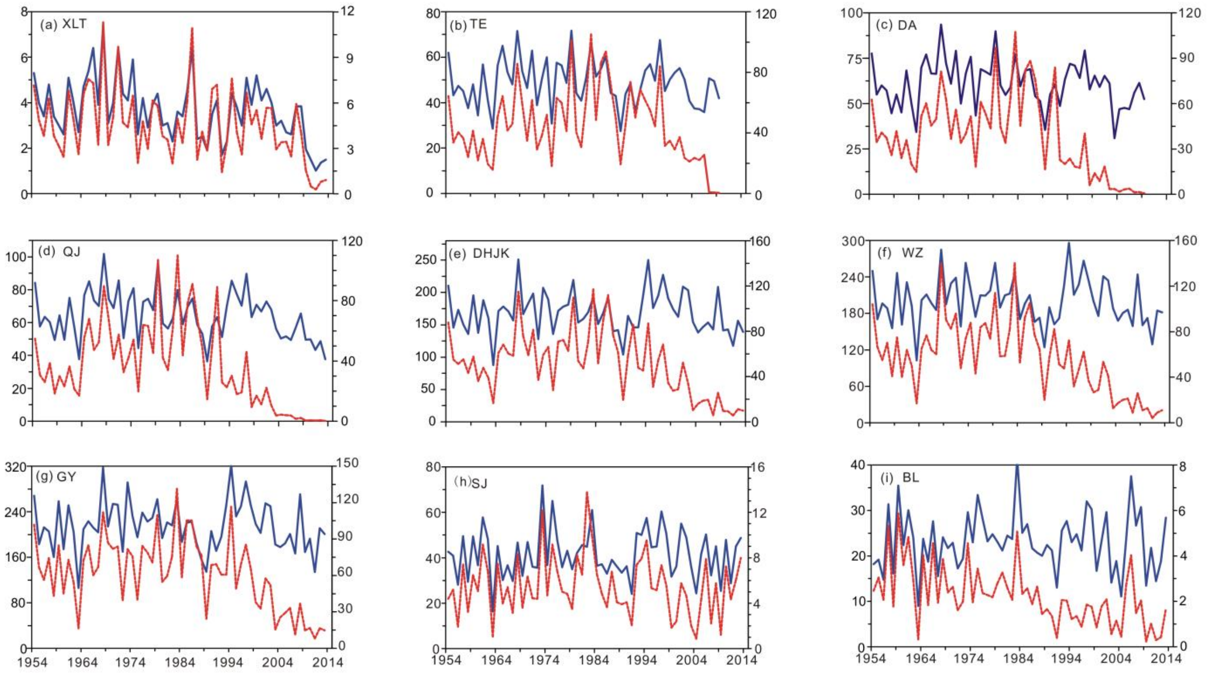

Figure S1. Annual averaged riverflow and sediment load data measured at the 9 analysed stations from 1954 - 2013 along the Pearl River. For station locations, please see Figure 1.

SUPPLEMENTARY TABLE (S1):

Table S1. Sediment Rating curves for Phase 1

Location	*a*	*b*	*r*	*p*
XLT	0.63	1.43	0.95	<0.001
TE	0.03	1.66	0.95	<0.001
DA	0.03	1.78	0.94	<0.001
QJ	0.02	1.88	0.82	<0.001
DHJK	0.007	1.74	0.89	<0.001
WZ	0.005	1.78	0.91	<0.001
GY	0.008	1.68	0.92	<0.001
SJ	0.01	1.61	0.95	<0.001
BL	0.05	1.33	0.98	<0.001
PR	0.009	1.59	0.86	<0.001

SUPPLEMENTARY TABLE (S2):

Table S2. Observed and predicted (with Phase 1 rating curves) sediment loads

Station	Phase	Period	Average sediment load (measured) (Mt/yr)	Average sediment load (calculated with Phase 1 rating curve) (Mt/yr)
XLT	1	1954-1979	5.08	5.09
	2	1980-1991	4.70	3.79
	3	1992-2006	4.51	4.16
	4	2007-2013	2.20	2.22
TE	1	1954-1979	45.37	44.65
	2	1980-1991	60.61	40.08
	3	1992-2006	40.77	39.44
	4	2007-2013	0.59	38.13
DA	1	1954-1979	45.37	44.99
	2	1980-1991	62.41	38.16
	3	1992-2006	13.67	38.46
	4	2007-2013	1.16	35.18
QJ	1	1954-1979	46.28	45.12
	2	1980-1991	60.87	35.11
	3	1992-2006	16.50	41.21
	4	2007-2013	0.87	24.38
DHJK	1	1954-1979	60.51	57.93
	2	1980-1991	70.31	48.91
	3	1992-2006	38.38	63.39
	4	2007-2013	11.04	45.39
WZ	1	1954-1979	71.89	71.59
	2	1980-1991	74.18	60.05
	3	1992-2006	38.06	76.09
	4	2007-2013	11.84	54.05
GY	1	1954-1979	70.97	69.29
	2	1980-1991	76.06	57.11
	3	1992-2006	52.73	74.25
	4	2007-2013	16.99	54.81
SJ	1	1954-1979	5.54	5.53
	2	1980-1991	6.19	4.99
	3	1992-2006	5.19	6.37
	4	2007-2013	4.97	4.99
BL	1	1954-1979	2.95	3.51
	2	1980-1991	2.27	3.62
	3	1992-2006	1.65	3.72
	4	2007-2013	1.02	3.33
PR	1	1954-1979	79.45	78.33
	2	1980-1991	84.53	65.72
	3	1992-2006	59.57	84.34
	4	2007-2013	22.99	63.14

SUPPLEMENTARY TABLE (S3):

Table S3. Sediment rating curves for PR for Phases 1-4

Phase	*a*	*b*	*r*	*p*
1	0.009	1.59	0.86	<0.001
2	0.007	1.69	0.75	=0.005
3	$2*10^{-5}$	2.58	0.91	<0.001
4	0.0009	1.84	0.96	<0.001
